# A prognostic model based on the Augmin family genes for LGG patients

**DOI:** 10.1038/s41598-023-34779-4

**Published:** 2023-05-09

**Authors:** Tao Wang, Senbang Yao, Siyu Li, Xichang Fei, Mingjun Zhang

**Affiliations:** Department of Oncology, The Second Affiliated Hospital of Anhui Medical University, Hefei, Anhui China

**Keywords:** Cancer genetics, CNS cancer, Tumour biomarkers

## Abstract

Gliomas are the most prevalent primary tumors in the central nervous system. Despite some breakthroughs in the treatment of glioma in recent years, survival rates remain low. Although genes of the Augmin family play a key role in microtubule nucleation, the role they play in gliomas is unclear. Transcriptome data were extracted from UCSC XENA and GTEx for low-grade glioma (LGG) and normal tissues, respectively. The protein interaction network associated with Augmin family genes was established using STRING and GeneMANIA databases. Enrichment analysis of gene-related functions and pathways was used to explore potential biological pathways and TIMER to assess immune cell infiltration. Regression analysis and Kaplan–Meier analysis were used to look at the clinical characteristics of the Augmin family genes and the association with the prognosis of patients with glioma. The results showed that the mRNA expression of Augmin family genes was significantly elevated in LGG tissues, except for HAUS7. Immunoregulation, cell cycle, apoptosis and other signaling pathways may be involved in the development and progression of LGG. Except for HAUS4 and HAUS7, the expression of all genes was positively correlated with immune cell infiltration. High expression of HAUS1, HAUS3, HAUS5, HAUS7, HAUS8 and low expression of HAUS4, HAUS6 in LGG was associated with poor prognosis. The risk models constructed based on the pivotal genes HAUS2, HAUS4 and HAUS8 were validated by nomogram and confirmed to be clinically useful for predicting the prognosis of LGG.

## Introduction

Glioma is the most common primary tumor in the central nervous system (CNS), accounting for approximately 80% of all types^1,2^. According to the 2016 World Health Organization (WHO) classification of CNS tumors, the majority of grade II and III gliomas are LGG including diffuse low-grade and intermediate-grade gliomas, while grade IV is mainly composed of glioblastomas (GBM) ^3^. In 2021, a new version of the classification of CNS tumors published by WHO added or established several molecular genetic features compared to the previous version (2016 version), which include ATRX, TPP53, CDKN2A/B and chromosomes Chr 7 and Chr 10 as well as the status of the transcription factor TERT^4^, which makes the classification of gliomas more specific and detailed. Gliomas are currently treated with surgery, radiation therapy, chemotherapy, immunotherapy, and targeted therapy^5–7^. Despite the many therapeutic options and multimodal treatment strategies available to us, the prognosis of glioma patients does not appear to have improved significantly. The prognosis for LGG is dismal due to unavoidable progression^8^, Only 5% of GBM patients will live beyond five years, with a median life expectancy time of only 12–14 months^9–11^. For this reason, in-depth analysis of glioma pathogenesis and to improve glioma treatment and diagnosis, molecular markers need to be explored.

Normal cell division depends on the proper assembly and function of the spindle^12,13^, and its assembly is driven by nucleation mechanisms, mainly including centrosome nucleation, chromatin-mediated nucleation, and Augmin complex-mediated nucleation^14,15^. The Augmin protein complex consists of eight subunits, which are: HAUS1 (Ccdc5), HAUS2 (Cep27), HAUS3 (hDgt3), HAUS4 (C14orf94), HAUS5 (hDgt5), HAUS6 (hDgt6), HAUS7 (UCHL5IP) and HAUS8 (Hice1). Augmin recruits γ-microtubulin (called γ-TuRC or γ-microtubulin ring complex) through its HAUS6 subunit to nucleate microtubules within the spindle^16^, and microtubule levels within the spindle are subsequently reduced when any of the subunits of Augmin are mutated or knocked out^17,18^, In a mouse embryo study, it was found that knocking out the HAUS6 gene in mouse apical progenitor cells resulted in spindle defects in mice, which caused massive apoptosis and eventually led to the termination of brain development^19^. This suggests that Augmin is essential for spindle formation and mitosis in mouse apical progenitor cells and for mouse brain development. In recent years, mutations in HAUS3 have been found in breast cancer^20^, and some scholars have knocked down Haus3 in hepatocellular carcinoma cell lines, which resulted in G2/M phase block in cancer cells and even inhibited tumor growth in vitro and in vivo^21^. Currently, the characterization of Augmin family-related genes in gliomas has not been systematically described.

In this study, we used several public databases to systematically interpret the expression pattern, function and clinical significance of Augmin family gene members in LGG, and developed a risk profile based on Augmin family to determine which patients are at high and low risk, with significant differences in genomic alterations, and prognosis between the two groups. We hope that our study can help clinicians to increase the accuracy of glioma prognosis prediction and select appropriate treatments and strategies.

## Materials and methods

### Data collection

RNAseq data from TCGA and GTEx were processed into TPM using the Toil program^22^, with data from UCSC XENA (https://xenabrowser.net/datapages/)^23^. The data was taken from 529 LGGs in TCGA and 1152 corresponding normal tissues in GTEx (paracancer samples lacking LGG in TCGA). The mRNAseq 325 dataset (TPM) and corresponding clinical data were downloaded from the Chinese Glioma Genome Atlas (http://www.cgga.org.cn) database^24,25^, and 182 LGG sample data were obtained from it. Cases with incomplete or missing data were removed from further analysis.

### HPA database

The Human Protein Atlas is an open database that uses imaging of antibodies, proteomics of mass spectrometry, and transcriptomics to map all the proteins in human cells, tissues, and organs^26^. This method was used to measure the expression of genes in tissues from the Augmin family.

### cBioPortal database

The cBioPortal (https://www.cbioportal.org) is an open web platform that contains data from 126 tumor genomic studies based on TCGA^27^. We mainly used this database to analyze the genetic variation of Augmin family genes.

### STRING database

STRING is a database that can be searched online for known and used to predict interactions between proteins, which currently contains 24,584,628 proteins from 5090 organisms^28^. We used this database to depict the protein–protein interaction (PPI) network of the Augmin gene family.

### GeneMANIA database

At the University of Toronto, GeneMANIA (http://www.genemania.org) is being actively developed, and other genes related with the input genes are discovered using association datasets (protein-gene interactions, pathway co-expression, co-localization, and structural domain similarities in proteins)^29^. In this study, we examined the pathways, co-expressions, and physical interactions of the Augmin family of genes.

### TIMER database

TIMER is a web server for analyzing tumor-infiltrating immune cells in depth. The TIMER method can measure the amount of six immunological infiltrates^30,31^. It was utilized to investigate immune cell infiltration in the LGG.

### Quantitative real-time PCR (qPCR) assay

Eight LGG tissues and normal brain tissues (from patients with traumatic brain hemorrhage) were collected by neurosurgeons of the Second Affiliated Hospital of Anhui Medical University from April 2022 to December 2022, and total RNA was extracted and stored in the refrigerator at – 80 °C. Tissue lysis, total RNA extraction, cDNA synthesis, and real-time quantitative PCR were performed using Easyzon Reagent (EnzyArtisan, China), HyperScript III RT SuperMix for qPCR with gDNA Remove (EnzyArtisan, China), and Universal SYBR qPCR Mix (EnzyArtisan, China). The primer design is shown in Table 1.

**Table 1:** HAUS2, HAUS4, HAUS8 and GAPDH primers for qRT-PCR.

Gene	Forward	Reverse
HAUS2	5′-GCTGGAGTTGGCTGTGACTTT-3′	5′-CTTTGCTAAAGCCTGGTTCATTT-3′
HAUS4	5′-GAGGACCTGTTACAGAACCCATAC-3′	5′-GCTTAAGCCACTCTCATCCACAT-3′
HAUS8	5′-GTGCTGGACTTACTGAGCGAAC-3′	5′-GGTTTGCCAAGGCTGCCTCTTT-3′
GAPDH	5′-GTCTCCTCTGACTTCAACAGCG-3′	5′-ACCACCCTGTTGCTGTAGCCAA-3′

### Statistical analysis

Analytical Statistics R version 4.1.0 was used to do the data analysis. P < 0.05 was chosen as the level of statistical significance. R is an open software programming language and operating system designed for statistical analysis, maps, and graphing^32^. Analysis of KEGG pathway enrichment^33^, immune cell infiltration, clinical correlation, and survival were conducted of Augmin family genes using R4.1.0. We also conducted univariate Cox analysis on training sets to identify Augmin genes that are associated with overall survival. A Cox model was developed for these genes. The model includes 3 genes and their characteristic risk scores were developed using a combining linearly the expression levels of expression levels of these 3 genes, risk score = (EXPORGENE1 × coefficientgene1) + (EXPORGENE2 × coefficientgene2) + (EXPORGENE3 × coefficientgene3). EXPORGENE1-3 are the TPM values for the expression of haus2, hasu4, haus8. Survival curves and subject operating characteristic curves (ROC) were used to assess the effectiveness of the model. The main r packages utilized in these analyses include ggplot2, clusterProfiler, limma, preprocessCore, BiocManager, estimate, caret, survminer, survival, glmnet, survROC, and pheatmap.

### Ethics approval and consent to participate

The study was approved by the Research Ethics Committee of The Second Affiliated Hospital of Anhui Medical University and followed the Declaration of Helsinki. All patients signed an informed consent form.

## Result

### Augmin family genes are aberrantly expressed in LGG

We analyzed the differential expression of eight genes of the Augmin family (HAUS1, HAUS2, HAUS3, HAUS4, HAUS5, HAUS6, HUAS7 and HUAS8) in LGG using R (ggplot2 package) (Wilcoxon rank sum test). Gene mRNA expression levels of the Augmin family were found to be significantly higher in LGG samples compared to normal brain tissue, except for HAUS7 (Supplementary Fig. 1A). We then further evaluated the correlation between Augmin family genes expression and the pathological stage of patients and found that the mRNA expression levels of HAUS1, HAUS2, HAUS3, HAUS5, HAUS7 and HAUS8 increased with tumor progression, except for HAUS4 and HAUS6, and there was a link between the expression of Augmin family genes and the stage of the disease (Supplementary Fig. 1B). As shown, HPA immunohistochemical staining also showed increased expression of Augmin family genes in LGG tissue (Supplementary Fig. 1C). These findings imply that genes from the Augmin family may play a role in tumor growth.

### Gene mutations and PPI networks of Augmin family genes in LGG

We focused examined the biological variation in the Augmin family genes in the TCGA LGG cohort using the cBioPortal database. We found that the mutation rates of HAUS1-HAUS8 were 0.7%, 0.7%, 0%, 2.5%, 2.1%, 2.1%, 2.5%, and 2.1%, respectively (Supplementary Fig. 2A). Survival curves (log-rank test) showed that cases with or without Augmin family genes alterations did not correlate with OS or DFS (Supplementary Fig. 2B). Using the STRING database, we next assessed the PPI network of Augmin family genes and the results showed 8 nodes and 28 edges of Augmin family genes (Supplementary Fig. 2C). We then used the GeneMANIA database to search for network-related genes, and the results showed that Augmin family protein network-related genes included POP1, ALG14, ACSF3, COG3, ATF4, YBX3, MLF1, NEDD1, TUBG2, VDAC1, RBM7, LTF, PIK3R2, WDR18, SPRYD7, HJURP, GLCE, MTMR12, and DNM3.Microtubule-related complexes and cell cycle control were the key roles of these genes (Supplementary Fig. 2D).

### Enrichment analysis of Augmin family genes

We performed Annotation of GO behavioral terms and analysis of pathways enriched in KEGG of Genes from the Augmin family and genes involved in protein networks (POP1, ALG14, ACSF3, COG3, ATF4, YBX3, MLF1, NEDD1, TUBG2, VDAC1, RBM7, LTF, PIK3R2, WDR18, SPRYD7, HJURP, GLCE, MTMR12, DNM3) using the clusterProfiler package (Supplementary Fig. 3A). The findings suggested that these genes were primarily concentrated at the BP level in ciliary basal body-plasma membrane docking, centrosome cycle control of G2/M transition of mitotic cell cycle, organelle localisation via membrane tethering, and so on. At the CC level it is mainly enriched in spindle, microtubule associated complex, microtubule, pericentriolar material. At the MF level it is mainly enriched in microtubule minus-end binding. We then used GSEA software (array of reference genes: kegg.v7.5.symbols.gmt) to analyze the enrichment of Augmin gene family members (Supplementary Fig. 3B). We found that Augmin gene family members are mainly enriched in cell cycle, apoptosis, proteasome, ubiquitin-mediated protein hydrolysis and other functions. HASU1, 2, 3, 5, and 6 were also enriched in T and B cell receptor pathways (although HAUS3, 5, and 6 p > 0.05). It was also abundant in a number of regulatory routes, including the P53, NOTCH, and TGF-BETA pathways. Further, members of the Augmin family gene were enriched in a variety of cancers, including prostate, small cell lung, pancreatic, and colorectal cancers. These findings support the hypothesis that the Augmin gene family is linked to carcinogenesis and progression.

### Infiltration of Augmin family genes by immune cells in LGG

**Figure 1:**
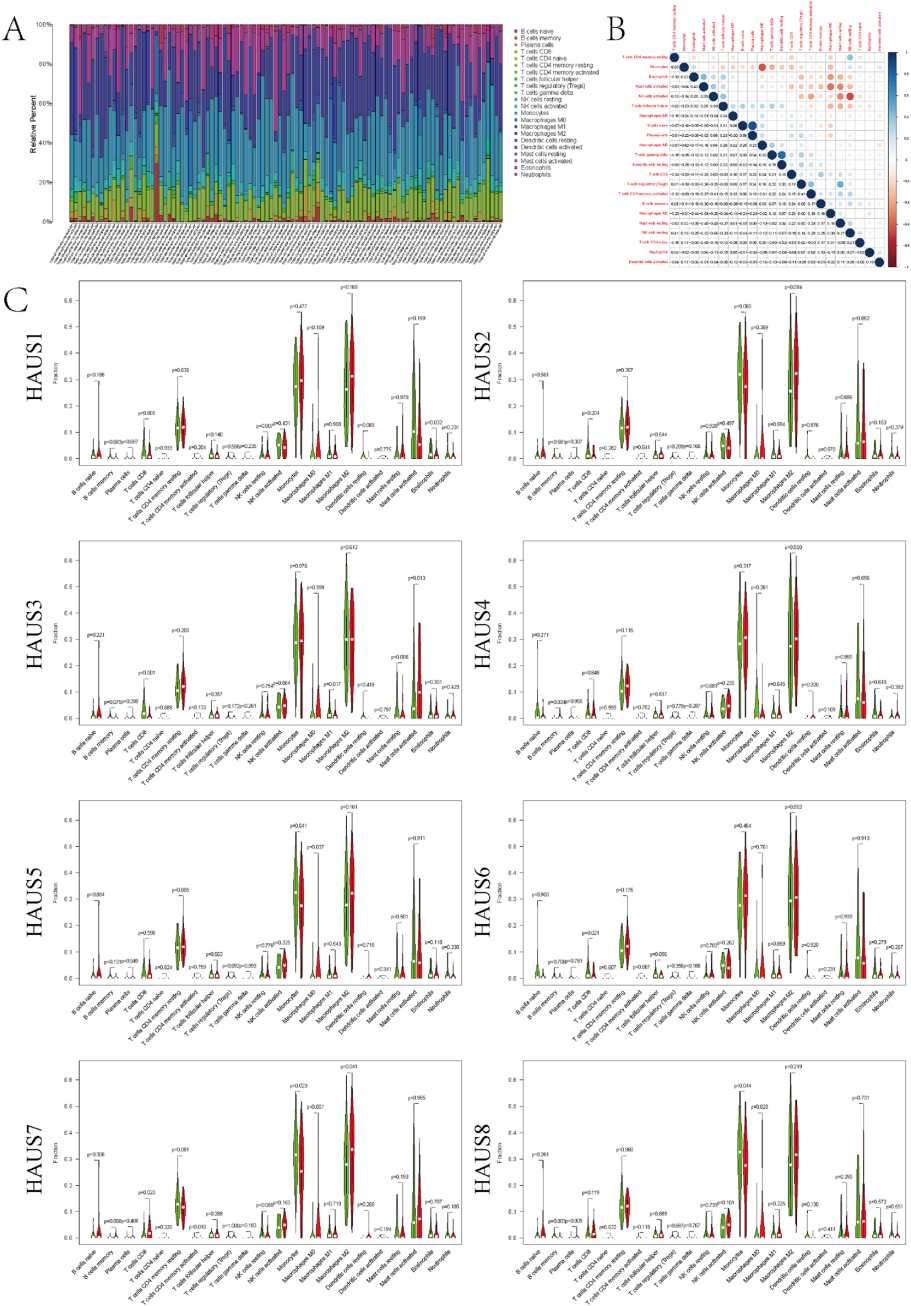
Immune infiltration of the Augmin family genes. (**A**) Proportion of 22 immune cell species in tumor samples from LGG patients. (**B**) Correlation between 22 types of immune cells in LGG samples. (**C**) Differences in 22 immune cells between high and low expression groups of Augmin family genes.

**Figure 2:**
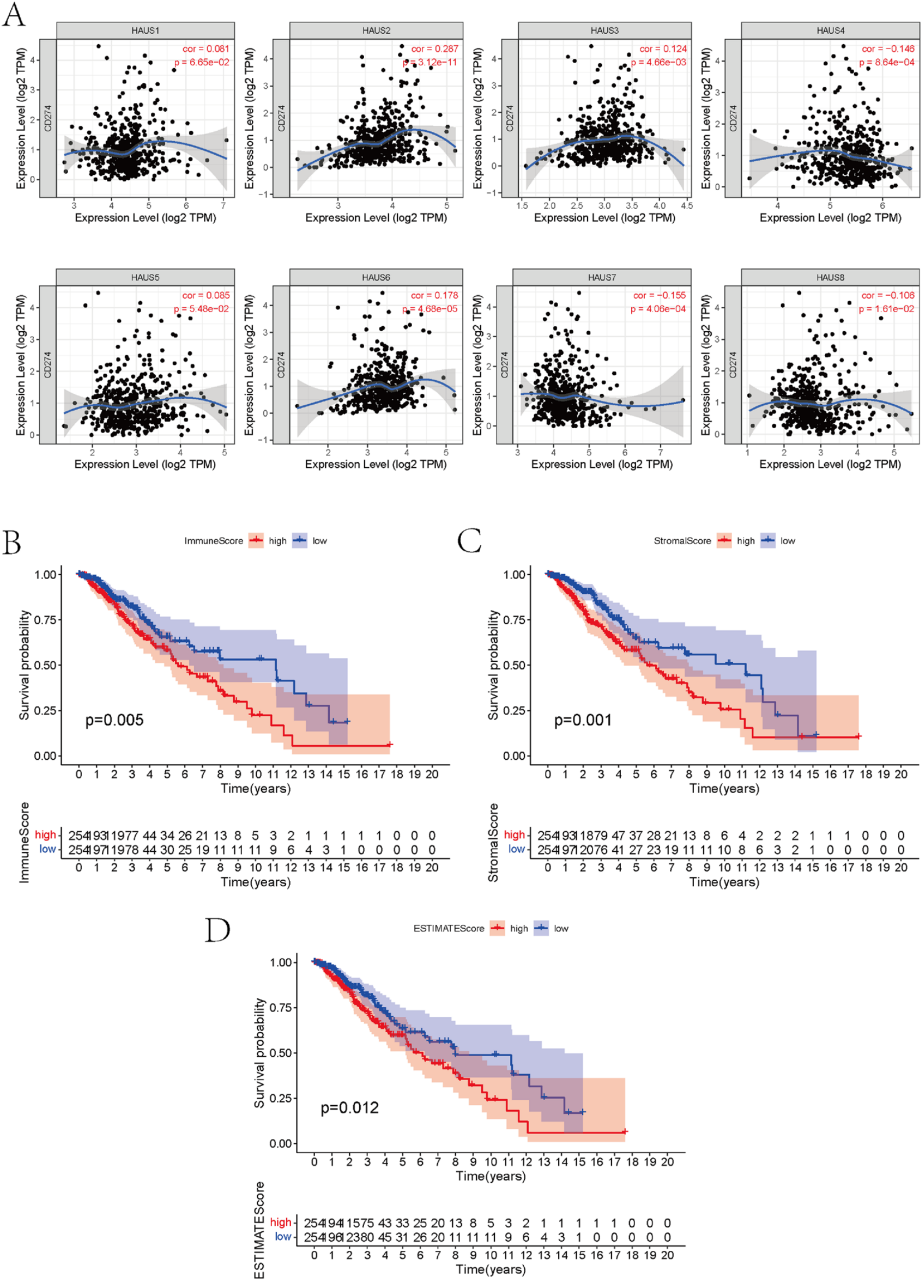
Immune infiltration of the Augmin family genes. (**A**) Correlation between Augmin family expression and PD-L1 (TIMER). (**B**–**D**) Survival analysis showed the relationship between immune cell content, stromal cell content, and their total content and survival of LGG patients.

Given the results of GSEA enrichment analysis, Augmin family genes may be related to immune regulation, and in addition to the critical function of immunity inside the tumor microenvironment, we analyzed the relationship between Augmin family genes and immune cells using the TIMER (Supplementary Fig. 4). The infiltration of CD8+ T Cell, B Cell, CD4+ T Cell, Neutrophil, Macrophage, and Dendritic Cell was strongly linked with the expression of HAUS1 and HAUS6 as seen in the figure. HAUS2 expression was positively correlated with the infiltration of all the above cells, except for the CD4+ T cells. HAUS3 was positively correlated with the infiltration of the above cells, except for Neutrophil, Dendritic Cell. HAUS4 was shown to be adversely linked with CD8+ T cell and neutrophil infiltration, but not with other cell infiltration (all p > 0.05). HAUS5 was shown to be negatively connected with CD8+ T cell infiltration and favorably correlated with the other cells indicated. HAUS7 was negatively correlated with infiltration of CD8+ T cells only, but not with other immune cells (all p > 0.05). Except for CD8+ T cells, HAUS8 was positively linked with infiltration of the aforementioned cells. These results show a strong positive correlation between these Augmin family genes, which may contribute to tumor progression, and immune cell infiltration of LGG. We then used the CIBERSORT algorithm to analyze the proportion of immune cell infiltrated subtypes to further confirm the role of Augmin family genes on the tumor microenvironment. In the LGG samples we constructed a profile of 22 of the immune cells (Fig. 1A) and calculated the correlation between these immune cells (Fig. 1B). We next looked at the alterations in 22 immune cells from high to low Augmin family gene expression groups one by one, discovering that the discrepancies were often in macrophage types. (Fig. 1C). We were perplexed by oncogenes that promote immune cell penetration, therefore we looked into the Augmin family genes and PD-L1 interaction once again (Fig. 2A), and the findings revealed that the invocation of HAUS1, HAUS2, HAUS3, HAUS5, HAUS6 (HAUS1, HAUS5 P > 0.05) was positively correlated with the expression of PD-L1, and HAUS4, HAUS7, HAUS8 showed a negative correlation of expression with PD-L1. We then performed survival analysis of LGG patients with different immune microenvironments (Fig. 2B–D), and as shown in the figure patients with lower immune cell content had better OS than those with higher immune cell content. The foregoing findings imply that the Augmin family genes may play a role in the immune regulation of LGG.

### Survival analysis and clinical correlation analysis

**Figure 3:**
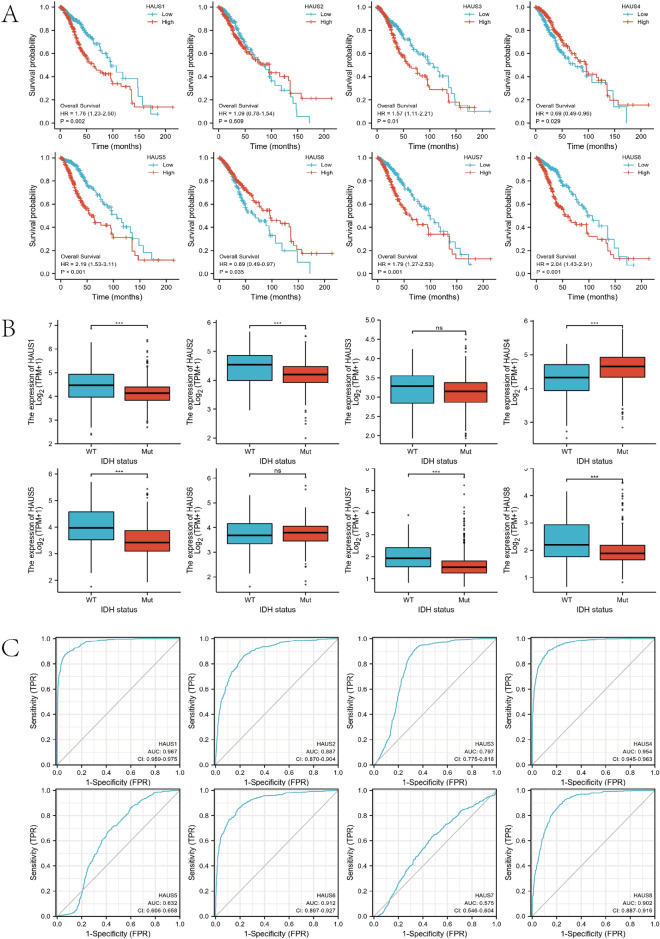
Augmin family genes survival analysis and clinical correlation analysis. (**A**) Survival analysis showed the association between high and low expression of Augmin family genes and survival of LGG patients. (**B**) Relationship between Augmin family genes expression and IDH wild-type and mutant phenotypes. (**C**) ROC curves for the Augmin family genes (*P < 0.05, **P < 0.01, ***P < 0.001).

The survival package was then used to investigate the link between the Augmin family genes and the overall survivorship of LGG patients (Fig. 3A). The research found that expression levels of the Augmin family genes HAUS1, HAUS3, HAUS4, HAUS5, HAUS6, HAUS7, and HAUS8 was all related with survival rates of glioma patients. Low expression of HAUS4 and HAUS6 and high expression of HAUS1, HAUS3, HAUS5, HAUS7, HAUS8 may be risk factors for poor prognosis in glioma patients. The expression of HAUS1, HAUS2, HAUS5, HAUS7, and HAUS8 was considerably greater in the IDH wild type than in the mutant, according to clinically correlation analysis (Fig. 3B). We then performed ROC analysis of the Augmin family genes. The outcomes revealed that the AUC (area under the curve) values of the ROC curves for HAUS1, HAUS2, HAUS3, HAUS4, HAUS6, and HAUS8 were > 0.75, which showed a high level of accuracy in forecasting LGG patients' prognosis (Fig. 3C).

### Construction of Augmin signature in TCGA LGG cohort

**Figure 4:**
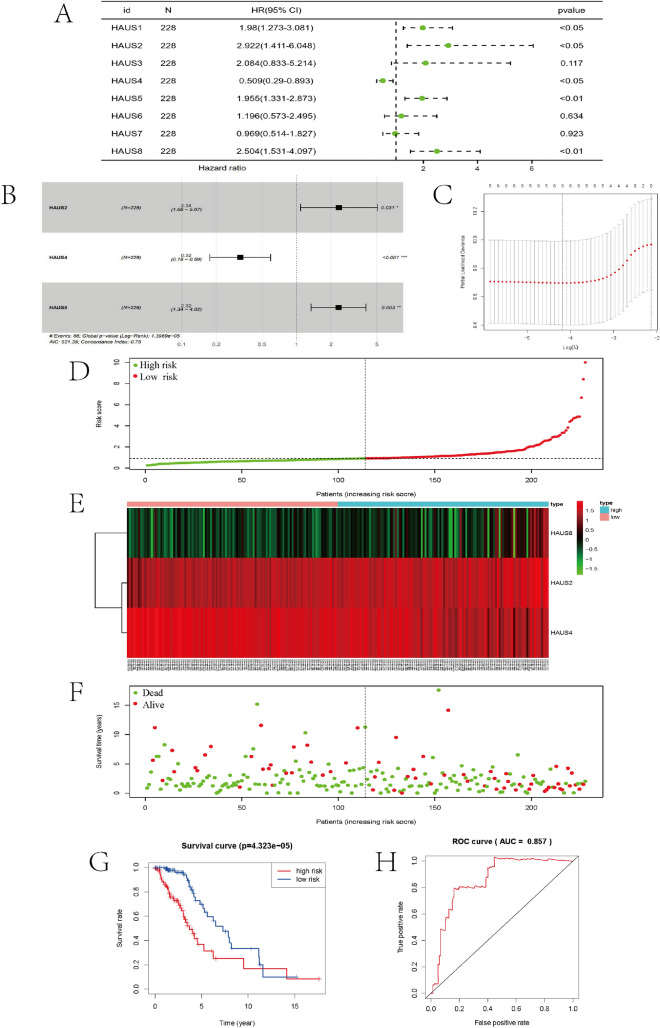
TCGA training cohort analysis. (**A**) Single-factor COX analysis of the Augmin family genes. (**B**) Multifactorial COX analysis of three candidate genes. (**C**) The most proper log (lambda) value in the LASSO model. (**D**) Patients were ranked by risk value. (**E**) Heat map of the expression of three candidate genes. (**F**) Survival status of low-risk and high-risk patients. (**G**) Survival analysis of low- and high-risk patients. (H) ROC curve.

**Figure 5:**
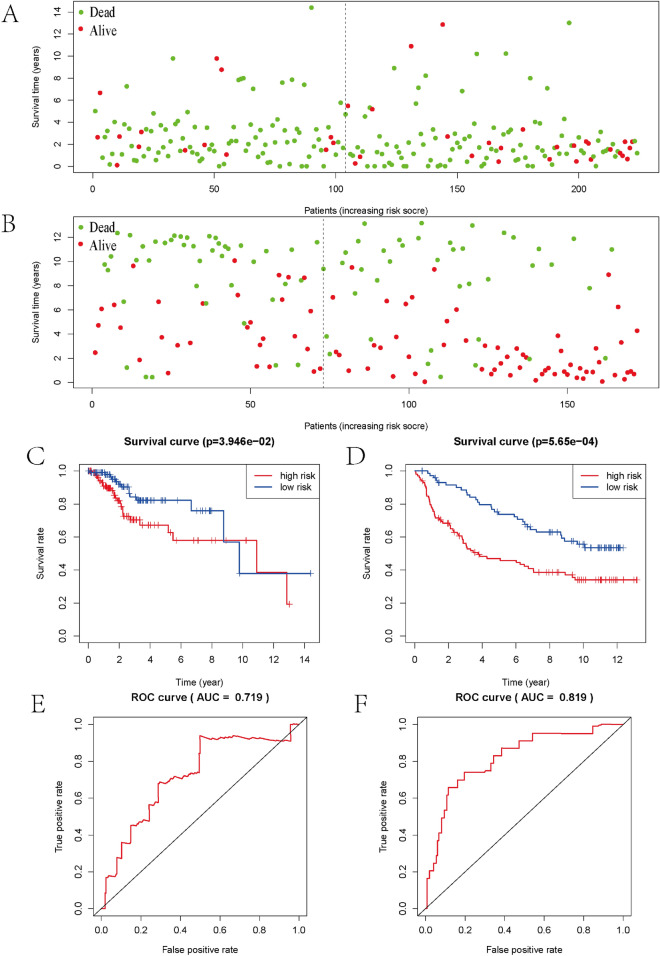
TCGA internal test cohort and CGGA external verification cohort analysis. (**A**,**B**) Survival status of high- and low-risk patients in the TCGA internal test cohort and the CGGA external validation cohort. (**C**,**D**) Survival analysis of high- and low-risk patients in the TCGA internal test cohort and the CGGA external validation cohort. (**E**,**F**) ROC curves for high- and low-risk patients in the TCGA internal test cohort and the CGGA external validation cohort.

Following that, we divided the LGG cohort in the TCGA database into two parts: a training cohort with 228 patients and a test cohort comprising 224 patients for internal test. For external validation, 172 samples from of the CGGA database were utilized. In the training cohort, the linkage between members of the Augmin family genes and overall survival of LGG patients was investigated using univariate Cox linear regression. We found that five genes, HAUS1, HAUS2, HAUS4, HAUS5, and HAUS8, were shown to be substantially linked to overall survival (p < 0.05) (Fig. 4A), and their HR values were greater than 1 except for HAUS4. We next used lasso linear regression to eliminate redundant genes before doing multiple regression model Cox analysis on the remaining genes in this prognosis-related gene set and modeled the risk scores (Fig. 4B,C). The 3 genes of the model are -haus2, haus4, haus8 and the following is the formula: risk score = 0.851950haus2 + (− 1.130684) * haus4 + 0.841221*haus8. Based on the median risk score, individuals in the training group got sorted into low—and high groups (Fig. 4D), and the expression of the three prognostic genes grew as the danger score grew (Fig. 4E). High-risk LGG patients tend to have poorer survival times than low-risk individuals (Fig. 4F). In the training cohort, Kaplan–Meier survival analysis revealed that OS was considerably better in the low-risk group than in the high-risk group (Fig. 4G). Moreover, subject operating characteristic (ROC) curve analysis revealed strong predictive power of our risk score model in both training cohorts (AUC = 0.857) (Fig. 4H). To improve the credibility of our risk model, we subjected the TCGA internal test cohort and the CGGA external validation cohort to the same analysis described above, and the conclusions obtained were largely consistent with the above. Individuals with low-risk ratings had a higher chance of surviving than those with high-risk ratings (Fig. 5A,B), and the low-risk group’s OS beat the high-risk group, according the Kaplan–Meier survival analysis (Fig. 5C,D), with ROCs of 0.719 and 0.819 for the TCGA internal test cohort and CGGA external validation cohort, respectively (Fig. 5E,F).

### The development and validation of a nomogram for prognosis evaluation

**Figure 6:**
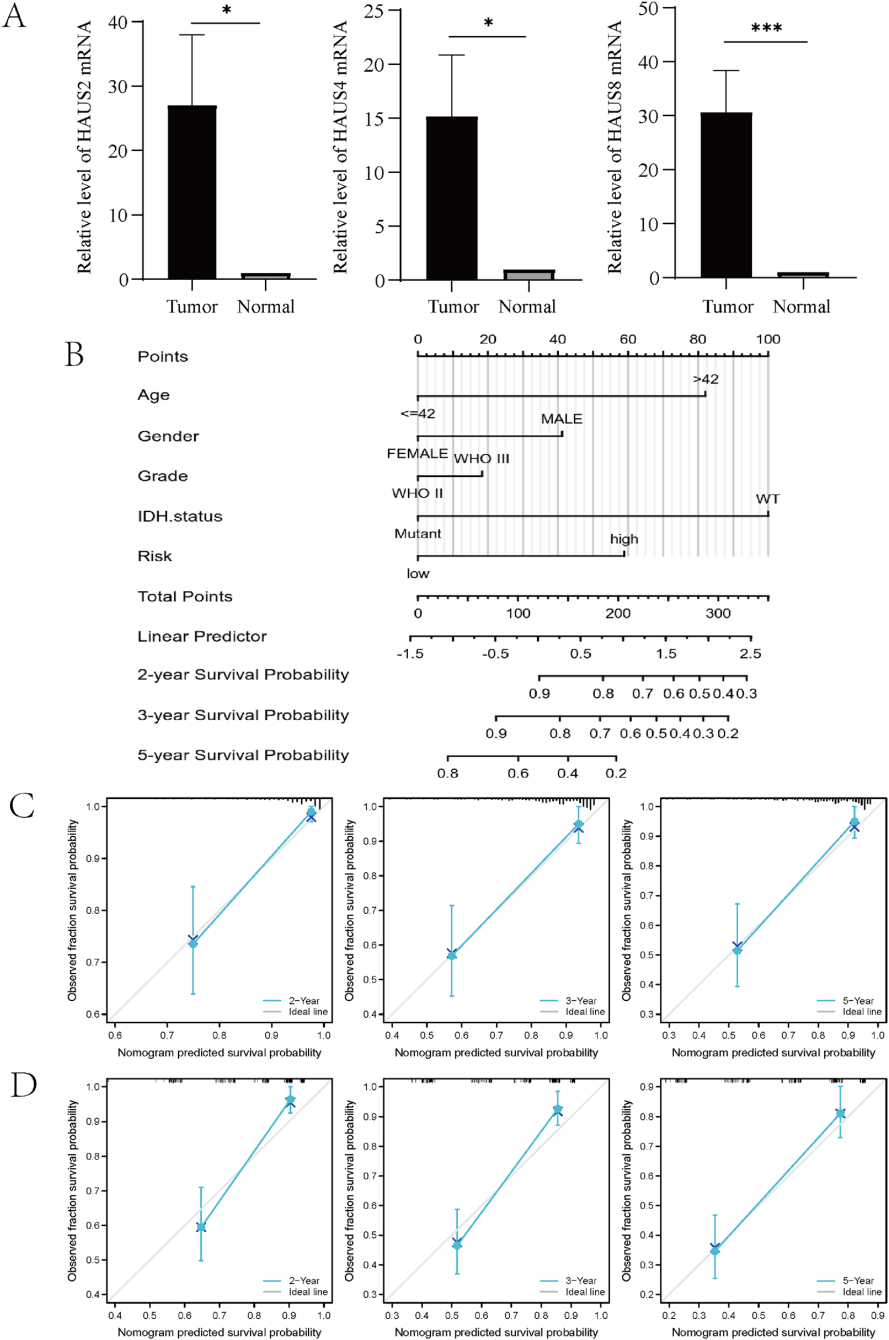
Creation and validation of Nomograms predicting 2-, 3-, and 5-year OS of LGG sufferers. (**A**) Relative mRNA expression of HAUS2, HAUS4 and HAUS8 in LGG tissues and normal brain tissues. (**B**) TCGA training cohort 2-, 3-, and 5-year OS prognostic nomograms. (**C**) Nomogram calibration plots for TCGA internal test cohorts for 2-, 3-, and 5-year OS. (**D**) Nomogram calibration plots for CGGA external validation test cohorts for 2-year, 3-year and 5-year OS (*P < 0.05, **P < 0.01, ***P < 0.001).

The mRNA expression of risk genes HAUS2, HAUS4 and HAUS8 in LGG tissue and normal brain tissue was verified in clinical samples by using qPCR. The results confirmed that the mRNA expression of HAUS2, HAUS4 and HAUS8 in clinical samples was higher in LGG than in normal tissues (Fig. 6A). To verify our forecasting model's independence, we included clinicopathological characteristics such as age, gender, histological grading, IDH status, and risk score in the analysis, and to see if Augmin signature was an independent predictive predictor, we utilized univariate and multivariate COX analyses. Multivariate COX analysis showed that the risk score model was shown to be substantially linked to overall survival, with HR = 2.044 in the TCGA training cohort (Table 2, 95% confidence interval [CI] = 1.140–3.666; p = 0.016) and HR = 1.588 in the CGGA validation cohort (Table 3, 95% confidence interval = 1.005–2.508; p = 0.048). We then built a Nomogram using data from the TCGA training set cohort, and we included age, gender, staging, IDH status, and risk score into the scoring criteria to arrive at a total score to reflect the OS at 2, 3, and 5 years (Fig. 6B). To validate the validity of the Nomogram, we drew calibration plots using the TCGA internal test cohort and the CGGA external validation cohort. For all cohorts, there was good agreement between the Nomogram's OS forecast and actual observation at 2, 3, and 5 years (Fig. 6C,D).

**Table 2:** Univariate and multivariate COX analysis of TCGA training cohort.

Characteristics	Total (N)	Univariate analysis		Multivariate analysis	
		Hazard ratio (95% CI)	P value	Hazard ratio (95% CI)	P value
Age	228				
≤ 42	123	Reference			
> 42	105	2.884 (1.714–4.855)	< 0.001	2.766 (1.594–4.799)	< 0.001
Gender	228				
Female	105	Reference			
Male	123	1.191 (0.732–1.938)	0.481		
Grade	228				
WHO II	99	Reference			
WHO III	129	2.188 (1.290–3.712)	0.004	1.227 (0.690–2.182)	0.487
IDH.status	228				
Mutant	182	Reference			
WT	46	4.252 (2.554–7.077)	< 0.001	2.707 (1.506–4.864)	< 0.001
Risk	228				
Low	114	Reference			
High	114	2.773 (1.669–4.609)	< 0.001	2.044 (1.140–3.666)	0.016

**Table 3:** Univariate and multivariate COX analysis of CGGA external validation cohort.

Characteristics	Total (N)	Univariate analysis		Multivariate analysis	
		Hazard ratio (95% CI)	P value	Hazard ratio (95% CI)	P value
Age	172				
≤ 42	105	Reference			
> 42	67	1.558 (1.027–2.362)	0.037	1.055 (0.683–1.632)	0.808
Gender	172				
Female	66	Reference			
Male	106	0.635 (0.420–0.960)	0.031	0.562 (0.369–0.856)	0.007
Grade	172				
WHO II	98	Reference			
WHO III	74	3.579 (2.335–5.485)	< 0.001	3.007 (1.913–4.728)	< 0.001
IDH.status	171				
Mutant	127	Reference			
Wildtype	44	2.640 (1.703–4.093)	< 0.001	1.845 (1.160–2.932)	0.010
Risk	172				
Low	73	Reference			
High	99	2.131 (1.372–3.309)	< 0.001	1.588 (1.005–2.508)	0.048

## Discussion

The diffuse infiltrative growth of glioma combined with the specificity of the central nervous system makes treatment more difficult, and patients have a poor quality of life, short survival and poor prognosis^34^. Several molecular genetic features have been used clinically as prognostic predictors for glioma patients, for example, IDH mutation status and 1p/19q deletion are considered as better prognostic markers^3^, and the search for more appropriate predictive and therapeutic targets has far-reaching implications for improving the prognosis of glioma patients. Augmin family genes are essential for spindle nucleation, cell mitosis and neuronal development^19,35–37^. It has also been found that by targeting the VISA complex, haus8 improves the RRR-VISA-dependent antiviral signaling pathway^38^. Augmin family genes have been less studied in cancer, and it has been found that HAUS3 is mutated in breast cancer and HAUS3 is a poor prognostic factor in liver cancer^20,21^. This is the first study to look at the transcription of Augmin family genes in LGG patients and their function and significance, which will help to improve the treatment strategy for LGG patients and forecast a patient's prognosis more accurately based on the existing knowledge of LGG.

First, the results of expression differences by analyzing the samples in the database showed that there were significant differences in the expression of Augmin family genes between LGG tumor samples and normal tissue samples, and more surprisingly, the expression of HAUS1, HAUS2, HAUS3, HAUS5, HAUS7 and HAUS8 (more than half of the genes) increased with tumor progression, and immunohistochemistry of HPA also showed elevated expression of Augmin family genes in LGG tissues. These suggest that Augmin family genes play a vital part in the growth and development of LGG. We then constructed the PPI network and analyzed the related genes of the family. Based on these genes we explored the functions of Augmin family genes using GO and KEGG enrichment analysis and found that Augmin family genes are mainly enriched in functions such as centrosome cycle, spindle assembly, and control of the mitotic cell cycle's G2/M transition. Chromosomal instability leads to intercellular heterogeneity, which promotes tumor heterogeneity and drug resistance^39^, and whether Augmin family genes alter LGG heterogeneity and drug resistance needs to be investigated in depth. Drugs targeting spindle assembly are now widely used in the treatment of human tumors^40^. The results of a GSEA enrichment research indicate that this family genes were mainly enriched in cell cycle, apoptosis, P53, T cell receptor, B cell receptor, TGF-BETA, NOTCH and other signaling pathways. These findings demonstrate that genes in the Augmin family might be used as therapeutic targets.

Studies on the possible role of Augmin family genes in human LGG are scarce, and correlation analysis between Augmin family genes expression in LGG and immune cell infiltration has not been studied. We discovered that these genes were also rich in T and B cell receptor pathways in a prior functional enrichment investigation, so we used the TIMER database to find that all genes in this family except HAUS4 and HAUS7 showed positive correlation with the invasion of immune cells. Subsequently, these genes' expression was examined in connection to PD-L1, and it was discovered that they were positively linked with PD-L1 expression, so we speculate that these genes not only promote the infiltration of immune cells but also may lead to increased PD-L1 expression on the tumor cell's surface. The consequence of PD-1 binding to PD-L1 is apoptosis and the failure of activated immune cells, making the tumor microenvironment immunosuppressive^41,42^. The specificity of the neuroanatomy and the immunosuppressive nature of gliomas are such that patients have very limited efficacy from conventional chemotherapy and radiotherapy^43^. Therefore, it is necessary to further investigate the mechanisms of action of these genes in order to develop appropriate targeted drugs to eliminate their immunosuppressive effects and thus improve the survival and prognosis of LGG patients.

Next, we used qPCR to demonstrate that the expression of HAUS2, HAUS4, and HAUS8 in LGG tissues in clinical samples was higher than that in normal brain tissues. Considering the use of prognostic models in clinical practice, we developed predicted OS at 2, 3 and 5 years based on risk score models, clinical characteristics of LGG and pathological parameters (age, gender, histological grading and IDH status). The internal test cohort was obtained from TCGA and the external validation cohort was taken from CGGA. Our results show that the calibration of nomogram achieves high consistency in both the internal test cohort and the external validation cohort. Each LCG patient can generate an independent nomogram based on their own characteristics, fully reflecting the personalization of clinical applications. The calibration curves also verified that the column line plots had high accuracy.

With the rapid development of high-throughput technologies, bioinformatics is increasingly developed in various fields of medical research such as disease-causing gene search and effective drug target screening. The occurrence, early diagnosis and prognosis of diseases depend on the constantly updated and refined progress of bioinformatics. Mastering the usage of advanced bioinformatics databases has become an urgent need for biologists and medical practitioners, and its results have greatly contributed to basic medical research. Genetic exploration based on public databases has been widely used for clinical diagnosis, treatment and prognosis prediction, which will help to identify disease susceptibility genes, elucidate the molecular mechanisms of disease occurrence, and thus provide opportunities to develop therapies that target key pathways of disease occurrence. This study provides insight into the role of the Augmin gene family in the prognosis and immune microenvironment of LGG, based on existing public databases, and provides a reference for further research on the specific mechanisms of Augmin in the future. We drew a nomogram for predicting patient OS based on the risk model. Each sample can be calculated by software to obtain the corresponding OS, which is virtually free, portable and intuitive in clinical applications. However, the present study also has many shortcomings and limitations. For example, the mechanisms of Augmin family genes involved in LGG developmental progression, especially cell cycle transition and immune infiltration, remain to be studied and validated in further in vitro or in vivo experiments. Second, the prediction model needs to be validated and updated in future large-scale clinical trials.

## Conclusion

This work used a thorough bioinformatics analysis to investigate the function of the Augmin family genes and its relationship with LGG. Augmin may be a viable target and prognostic biomarker for LGG patients, according to our findings. Additional molecular tests are needed, however, to better corroborate the findings of this study and to make Augmin family genes more clinically useful in LGG patients.

## Supplementary Information

Supplementary Figures.

## Data Availability

The original contributions presented in the study are included in the article or Supplementary Material; further inquiries can be directed to the corresponding author.
