# Supplementary material for: A prognostic model based on the Augmin family genes for LGG patients
Source: Sci Rep. 2023 May 9;13:7520. doi: 10.1038/s41598-023-34779-4 (PMC10170088; doi:10.1038/s41598-023-34779-4)
Supplement: Supplementary file 1 — Supplementary Figures. [file 41598_2023_34779_MOESM1_ESM.pdf]

A prognostic model based on the Augmin family genes for LGG patients

Tao Wang[1#], Senbang Yao[1#], Siyu Li[1], Xichang Fei[1], Mingjun Zhang[1*]

[1] Department of Oncology, The Second Affiliated Hospital of Anhui Medical University, Hefei, Anhui, China.

*** Address correspondence to:** Mingjun Zhang, E-mail: zhangmjayd@126.com.
# Contributed equally.

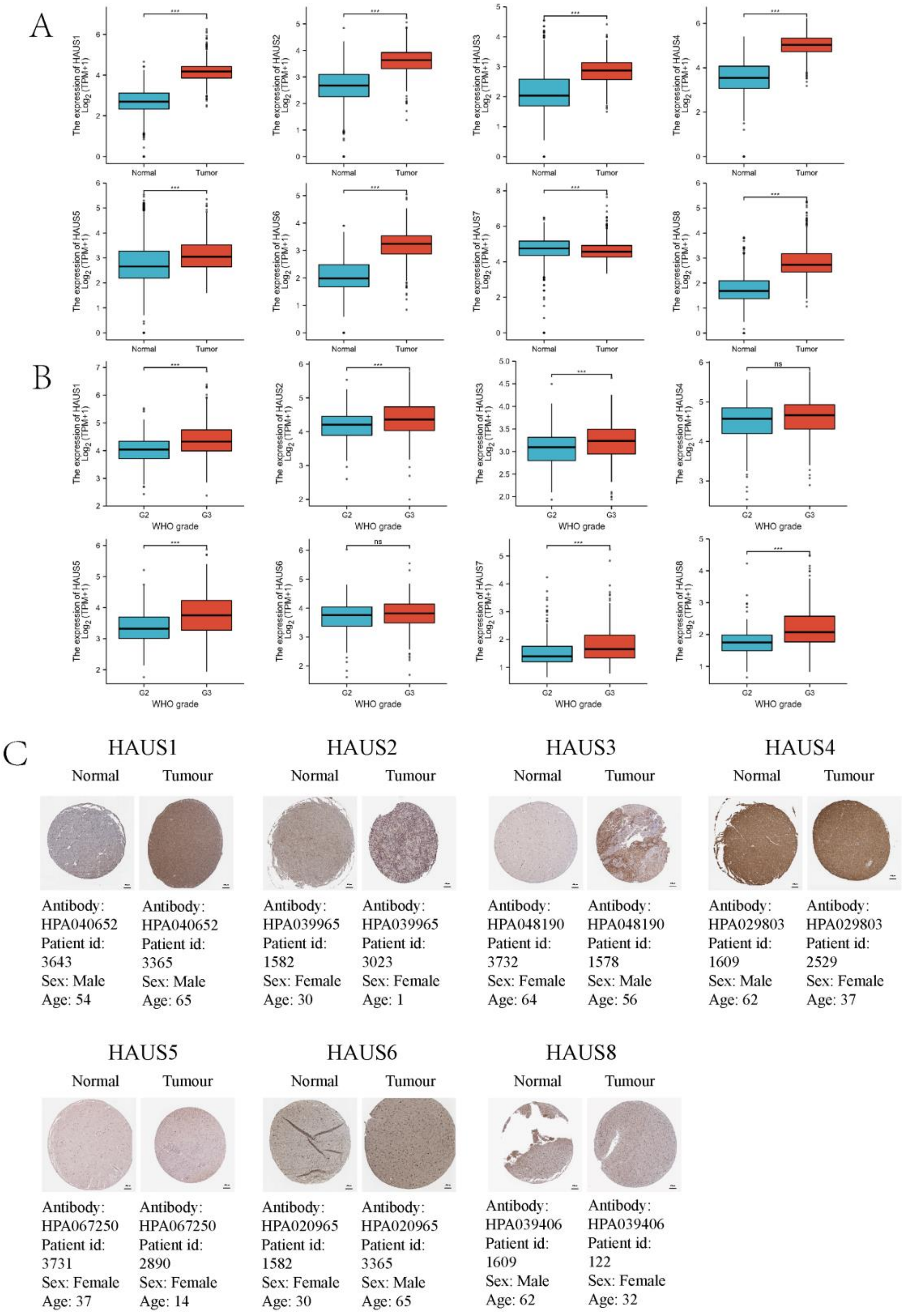

Supplementary Figure 1. Aberrant expression of Augmin family genes in LGG. (A) Differential expression of Augmin family genes between LGG and matched normal as well as GTEx. (B) Relationship between Augmin family gene expression and pathological grading. (C) Augmin family protein expression levels in normal and tumor tissues, image from HPA database. (HAUS7 assay is not available in HPA glioma *P<0.05 **P<0.01 ***P<0.001).

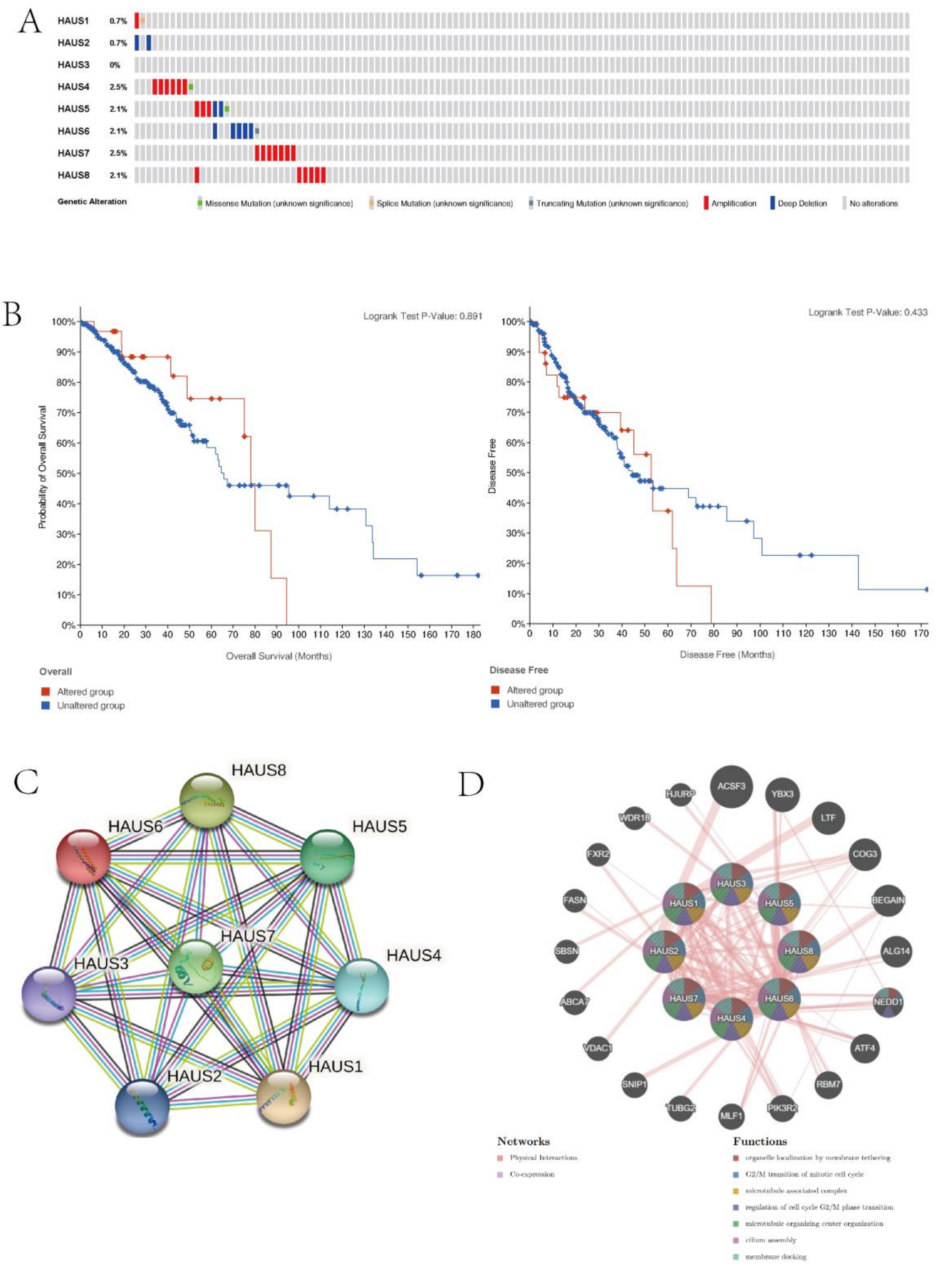

Supplementary Figure 2. Augmin family mutations in LGG, PPI network and related genes. (A-B) Augmin family gene mutations in LGG and the relationship between gene mutations and survival of LGG patients, image from cBioPortal database. (C) PPI network of Augmin family genes. (D) Augmin family protein network-related genes.

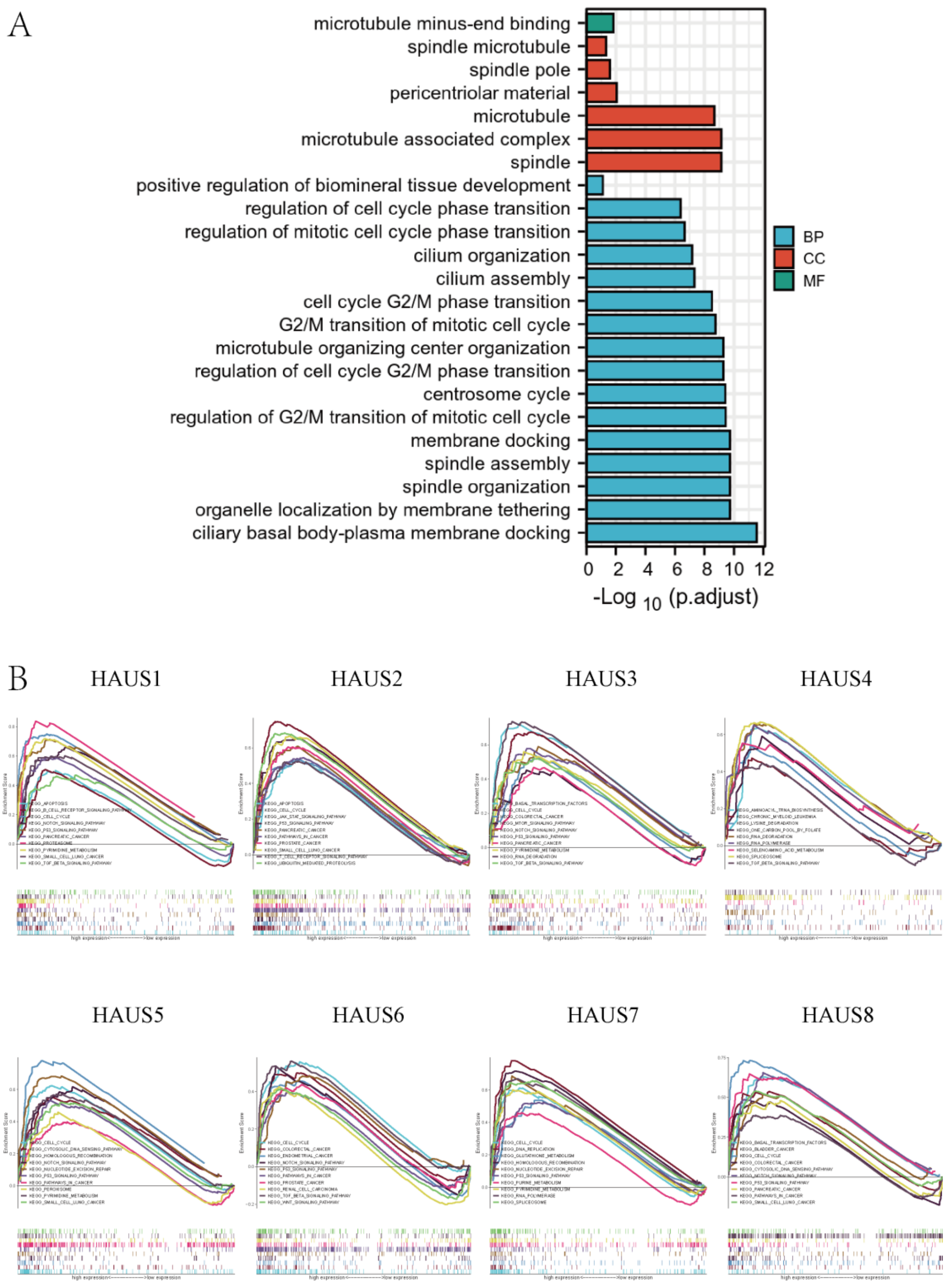

Supplementary Figure 3. Results of enrichment analysis of Augmin family genes. (A) GO functional annotation and KEGG pathway enrichment analysis of Augmin family genes and protein network-related genes. (B) GSEA analysis of Augmin family genes.

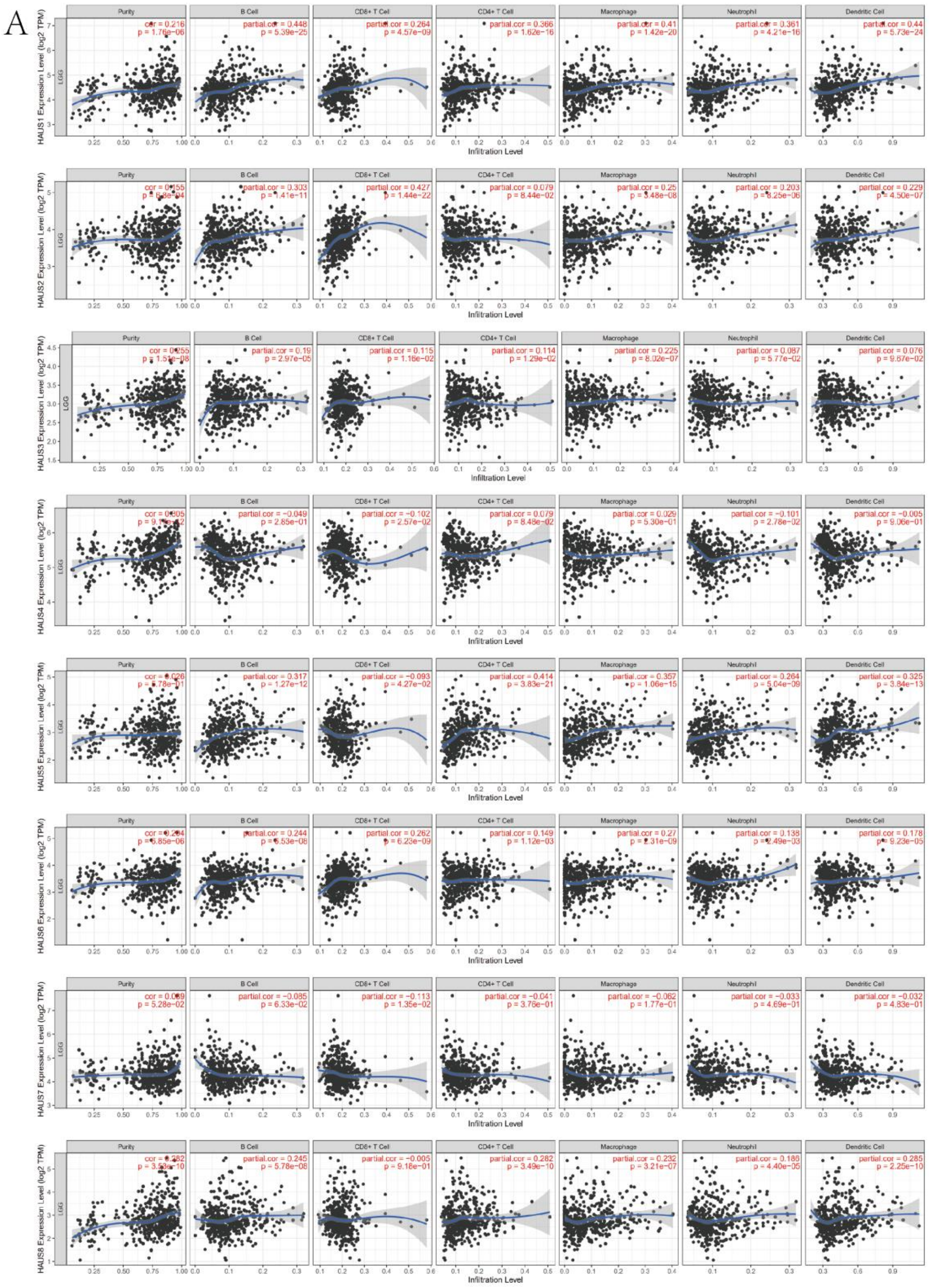

Supplementary Figure 4. Correlation between Augmin family gene expression and eight types of immune cell infiltration.
